# Impact of γ-chain cytokines on EBV-specific T cell cultures

**DOI:** 10.1186/1479-5876-8-121

**Published:** 2010-11-22

**Authors:** Anna Merlo, Riccardo Turrini, Cristina Trento, Paola Zanovello, Riccardo Dolcetti, Antonio Rosato

**Affiliations:** 1 University of Padova, Dept. of Oncology and Surgical Sciences, Via Gattamelata 64, 35128 Padova, Italy; 2 Department of Haematology, Imperial College, Du Cane Road, London, UK; 3 Istituto Oncologico Veneto IRCCS, Via Gattamelata 64, 35128 Padova, Italy; 4 CRO, Centro Riferimento Oncologico IRCCS, Via F. Gallini 2, 33081 Aviano, Italy

## Abstract

**Background:**

Recent preclinical adoptive immunotherapy studies in murine models prompt to employ "proper" rather than "as many as possible" antigen-specific T cells to gain better therapeutic results. Ideally, "proper" T cells are poorly differentiated *in vitro*, but retain the capacity to fully differentiate into effector cells *in vivo*, where they can undergo long-term survival and strong proliferation. Such requirements can be achieved by modifying culture conditions, namely using less "differentiating" cytokines than IL-2.

**Methods:**

To evaluate this issue in human T cell cultures, we exploited a well characterized and clinical-grade protocol finalized at generating EBV-specific CTL for adoptive immunotherapy. In particular, we studied the impact of IL-7, IL-15 and IL-21 compared to IL-2 on different aspects of T cell functionality, namely growth kinetics, differentiation/activation marker expression, cytokine production, and short-term and long-term cytotoxicity.

**Results:**

Results disclosed that the culture modifications we introduced in the standard protocol did not improve activity nor induce substantial changes in differentiation marker expression of EBV-specific CTL.

**Conclusions:**

Our data indicated that the addition of γ-chain cytokines other than IL-2 for the generation of EBV-specific T cell cultures did not produce the improvements expected on the basis of recent published literature. This fact was likely due to the intrinsic differences between murine and human models and highlights the need to design *ad hoc *protocols rather than simply modify the cytokines added in culture.

## Background

Infusion of antigen-specific T cells proved to be safe and effective against both virus infections (e.g., CMV [1]) and cancer, in particular melanoma and EBV-driven malignancies [2]. The vast majority of current protocols rely on the infusion of a high  number of effector cells that require long-term in vitro cultures, in  particular when dealing with Tumor Infiltrating Lymphocytes (TIL) or  clonal cultures. Consequently, this aspect implies labor-intensive and cost-ineffective procedures and, furthermore, has a potential negative impact on the characteristics of cells infused. Indeed, as advanced by Gattinoni and colleagues [3,4], long-term T cell cultures move toward a differentiated phenotype characterized by a high cytotoxic potential, but also a poor recirculation and *in vivo *expansion capability. These features are highlighted by a well-defined "marker expression signature", namely CD27^low/neg^, CD28^low/neg^, CD62L^low/neg^, CCR7^low/neg^, and CD57^high^. Thus, the new trend in adoptive cell therapy (ACT) focuses on the infusion of a more limited number of cells, but with the "proper" phenotype and functional characteristics, which can promote prolonged *in vivo *persistence and expansion, and induction of immunological memory to provide protection against possible relapses. The potentiality to expand and persist in the host also relies on the possibility for the infused cells to find an "immunological space" to colonize. This is "naturally" accomplished in Post Transplant Lymphoproliferative Disease (PTLD) after Haemopoietic Stem Cell Transplantation (HSCT), in which patients are immunocompromised due to the immunosuppressive regimens; in patients with other tumors, it has been achieved by chemotherapy and irradiation [5] or by immunodepleting (anti-CD45) antibodies [6]. In these conditions, infused T cells have a favourable environment with fewer competitors for and elevated availability of homeostatic cytokines (IL-7 and IL-15), and possibly less numerous T regulatory (Treg) populations.

Although much attention has been paid to shorten the generation protocols in the clinical settings, a stringent correlation between phenotype (and so differentiation) and outcome has been shown mainly in mouse models thus far [4,7,8], with few notable exceptions [9]. In this context, several reports have described the impact of different γ-chain cytokines on the differentiation status and functional properties of T-cell cultures *in vitro *and, more importantly, *in vivo*. Overall, they suggested that certain γ-chain cytokines, in particular IL-15 and IL-21, are superior to the commonly used IL-2 in maintaining a less differentiated phenotype of cultured T cells, thus possibly resulting in a better therapeutic activity. In this regard, the eradication of large established melanomas (approximately 50 mm^2 ^tumor area) was achieved by the infusion of as little as 5 × 10^5 ^IL-21 cultured T cells [7].

To explore this critical issue in human T cell cultures, we took advantage of a well established and clinical-graded protocol aimed at generating EBV-specific CTL for ACT. We slightly modified the protocol by adding to the cultures IL-7, IL-15 or IL-21 instead of IL-2. Moreover, we separated and maintained in parallel cultures CD4^+ ^and CD8^+ ^T cells to better discriminate the impact of the different cytokines on the two subsets. We therefore compared the proliferative potential, phenotype, cytokine production, and cytotoxic activity of effector cells obtained in different culture conditions. On the whole, addition of different cytokines did not produce any clear improvement or substantial differences between T cell lines. Therefore, to obtain more active T cells for therapy, we infer that several other conditions need to be optimized other than the use of different cytokines, namely *ad hoc *protocols able to appropriately balance the effector cell expansion and the timing of culture.

## Methods

### Lymphoblastoid cell lines (LCL)

EBV-transformed lymphoblastoid cells were generated from peripheral blood mononuclear cells of HLA-typed healthy donors using culture supernatant from the EBV-producing marmoset cell line B95.8 (American Type Culture Collection). Signed informed consent was obtained from the donors and the research protocol was approved by the institutional ethical review board of the Istituto Oncologico Veneto, in accordance with the ethical standards of Helsinki Declaration.

Cyclosporin A (CsA, Sandoz Pharmaceuticals AG; Cham, Switzerland) was initially added to the cultures to inhibit T cell growth (final concentration, 700 ng/ml). LCL were maintained in RPMI 1640 (Euroclone, Pero, Milan, Italy) supplemented with 10% heat-inactivated type AB Human Serum (HS, Lonza BioWhittaker; Basel, Switzerland), 1 mM Na Pyruvate, 10 mM Hepes Buffer, 2 mM Ultraglutamine (all from Lonza BioWhittaker), 1% Antibiotic/antimycotic (Gibco, Invitrogen Corporation), hereafter referred to as HS complete medium.

### Generation of EBV-specific CD4^+ ^and CD8^+ ^T-cell lines

EBV-specific T cells were established as previously described [10], with modifications. Briefly, PBMC were co-cultivated with irradiated (40 Gy) autologous LCL at a ratio of 40:1 in 24-well plates (Corning Incorporated; Corning, NY) in HS complete medium. PBMC were seeded at a concentration of 2 × 10^6 ^cells/ml and maintained at 37°C in a 6.5% CO_2 _humidified atmosphere. On day 10 and weekly thereafter, CTL were re-stimulated with irradiated LCL at a 4:1 ratio. Recombinant IL-2 (35 I.U./ml, Proleukin, Chiron Corporation; Emeryville, CA) or IL-7 (10 ng/ml; Peprotech; Rocky Hill, NJ) or IL-15 (10 ng/ml; Peprotech) or IL-21 (10 ng/ml; eBioscience; San Diego, CA) were added on day 14 and replenished every 2 days. On day 14, before cytokine addition, CD4^+ ^T cells were immunomagnetically sorted using the CD4^+ ^T cell Isolation Kit II (Miltenyi Biotec; Bergisch Gladbach, Germany), and both CD8^+ ^and CD4^+ ^T cells were cultured in parallel. At each subsequent re-stimulation with LCL, CD4^+ ^T cells were adjusted to 1.5 × 10^6 ^cells/ml and CD8^+ ^T cells to 2 × 10^6 ^cells/ml.

### Cytotoxicity assays

Cytotoxic activity of CD4^+ ^and CD8^+ ^T cells was determined in a standard 4-h ^51^Cr release assay, as previously reported [11]. Autologous LCL were used as target cells, while K562 cell line served as indicator of NK-like activity. All tests were carried out with an excess of unmarked ("cold") K562 (5:1 ratio between "cold" and "hot" target). Where indicated, CD4^+ ^T cells were pre-treated for 2 h at 37°C with either 20 μM brefeldin A (BFA, Sigma-Aldrich; St. Louis, MO) or 100 nM concanamycin A (CMA, Sigma-Aldrich) and assayed in the presence of the drugs. To assess calcium-dependence of cytolytic activity, 4 mM EGTA (Sigma-Aldrich) was added to the assay. For antibody blocking experiments, T cells were pre-incubated with 10 μg/ml of anti-FasLigand mAb (clone NOK-1; BioLegend; San Diego, CA).

### Flow cytometry

Surface markers were determined by staining with FITC- or PE-conjugated antibodies and the respective isotypes. CTL lines were stained with antibodies to CD3, CD16, CD56 (BD-Pharmingen; San Diego, CA), CD4 and CD8 (BD Biosciences; San Diego, CA), CCR7 (eBioscience), CD27, CD28, CD57, CD62L and CD127 (IL7Rα; BioLegend). Cells (2 × 10^5^) were washed with phosphate-buffered saline (PBS; Sigma-Aldrich) and re-suspended in 50 μl of staining solution (PBS, 3% FBS and 0,1% NaN_3_) containing an optimal concentration of antibody. After a 20-minute incubation in ice, cells were washed again and analyzed using a FacsCalibur (BD) flow cytometer. Flow cytometry data were analyzed with FlowJo software (Tree Star, Inc.; Ashland, OR).

### ELISA test

Cytokine ELISA tests were performed using Human TNFα Screening Set and Human IFNγ Screening Set (Thermo Scientific, Rockford, IL), according to the manufacturer's instructions. Briefly, 2 × 10^5 ^effector cells and 2 × 10^5 ^autologous LCL were seeded in 96-well round-bottom plates. Positive controls were represented by effector T cells incubated with PMA-ionomycin (40 ng/ml and 4 μg/ml, respectively; Sigma-Aldrich). Baseline cytokine production was determined in supernatants from unstimulated T cells, or LCL only. Cytokine secretion was measured after 5h-incubation.

### Outgrowth assay

Outgrowth assay was carried out as previously described [12]. Briefly, target LCL were seeded as replicates in U-bottom 96-well plates at doubling dilution, starting from 10^4 ^cells/well to 78 cells/well. T cells were added to half of the replicates at 10^4 ^cells/well in HS complete medium without IL-2. Plates were then incubated at 37°C in 6.5% CO_2 _and re-feeded weekly by replacing half of the medium. LCL outgrowth was scored after 4 weeks by visual examination with an inverted microscope. Results are expressed as the minimum number of LCL required for successful outgrowth in 50% of replicate wells.

## Results

### Analysis of *in vitro *growth kinetics

**Figure 1:**
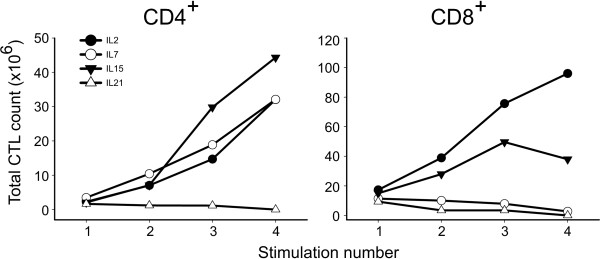
**Growth kinetics of CD4^+ ^and CD8^+ ^T cell lines**. The extrapolated mean total cell counts of CD4^+ ^(left) and CD8^+ ^(right) T cell lines cultured with IL-2, IL-7, IL-15 and IL-21 before each re-stimulation with LCL is represented. Figure shows mean values from at least two independent experiments.

To dissect the impact of different γ-chain cytokines on human T cell *in vitro *expansion, we took advantage of a well defined protocol aimed at generating EBV-specific T cells cultures [10,13]. First, we evaluated the proliferative potential of CTL lines cultured with IL-15, IL-7 or IL-21 in comparison to IL-2. Briefly, we seeded PBMC from healthy donors with autologous LCL without cytokine addition for the selection phase. Two weeks later, the expansion phase was started by supplying different cytokines to purified CD8^+ ^and CD4^+ ^T cells, to assess their proliferative response. As expected, we found that both CD8^+ ^and CD4^+ ^T cells grew vigorously when cultured with IL-2, although with differential magnitudes. In particular, CD4^+ ^T cells grew for a longer time (more than 14 weeks) in comparison to CD8^+ ^T cells, which disclosed an initial phase of logarithmic growth followed by a progressive reduction of their active proliferation after 3 to 7 re-stimulations (Figure 1 and data not shown). IL-15 produced a similar trend in CD4^+ ^and CD8^+ ^T cell growth and proved to be superior to other tested cytokines in inducing the expansion of both subpopulations, while IL-7 supported the expansion of CD4^+ ^T cells only, albeit at different degrees of magnitude for different donors. In deep contrast, IL-21 alone allowed survival but did not sustain the expansion of either subsets of T cells, in line with previously reported data [14-16].

### Assessment of phenotype

**Figure 2:**
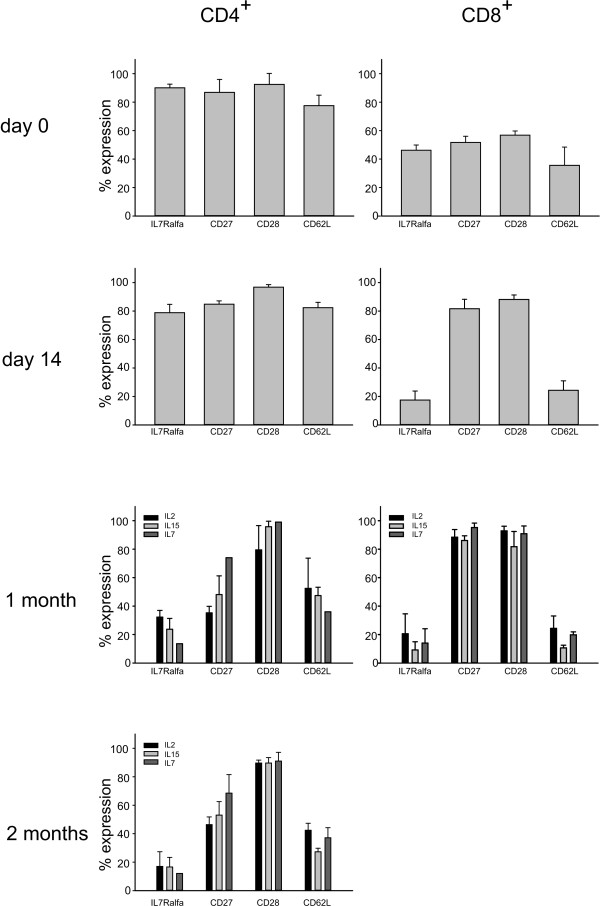
**Expression of maturation/differentiation markers**. Figure shows marker expression by CD4^+ ^and CD8^+ ^T cells at day 0, 14 (before separation and cytokine supply), 1 month and, for CD4^+ ^T cells only, 2 months. Figure shows mean +/- SD of 3 replicate cultures from 2 donors.

The use of different cytokines in culture could impact on differentiation, trafficking and functional properties of T cells, characteristics that have a counterpart on specific surface marker expression [3]. We therefore analyzed the expression of CD27, CD28, CD57, CD62L, IL7Rα,  and CCR7 at different time points during culture. We performed flow cytometry analysis at day 0 just before seeding, at day 14 before immunomagnetic separation and cytokine addition, and after 1 month of culture. At 2 months, phenotype of CD4^+ ^T cells only could be evaluated, since CD8^+ ^T lymphocytes did not proliferate so long. The phenotype of IL-21 T cells could not be determined due to the low number of lymphocytes obtained in these cultures. As shown in Figure 2, overall we found more pronounced differences in the phenotypic profile of CD8^+ ^and CD4^+ ^T cells prior to the addition of the various cytokines than after their supplement to cultures. Indeed, immediately after *ex vivo *collection, nearly all CD4^+ ^T cells expressed CD27, CD28, CD62L, IL7Rα, in comparison to only about 50% of CD8^+ ^T cells. Conversely, CD8^+ ^T cells tended to acquire CD27 and CD28 expression in culture, differently from what observed by Vanhoutte *et al. *[17], while IL7Rα and CD62L were poorly represented in this subset respect to the CD4^+ ^T cell counterpart. These latter cells, on the contrary, partly lost the CD27 expression during culture. The expression of CCR7, which appeared initially quite variable between CD4^+ ^and CD8^+ ^T cells, was lost by all T cell lines from the third week of culture and thereafter (data not shown); on the other hand, CD44 was expressed at high intensity in nearly all T cells for the entire period of culture (data not shown). CD57 expression was quite different between CD4^+ ^and CD8^+ ^T cells (4.35 +/- 3.44% versus 22.99 +/- 5.15% immediately after *ex vivo *collection, respectively); in fact, it was rapidly up-regulated and then lost by CD4^+ ^T cells, while retained by CD8^+ ^T cells (data not shown). Finally, after 1 month of culture the phenotypic profile tended to stabilize and did not further modify substantially at least for the CD4^+ ^T cell subset, the only one that could be tested.

### Evaluation of cytokine production

**Figure 3:**
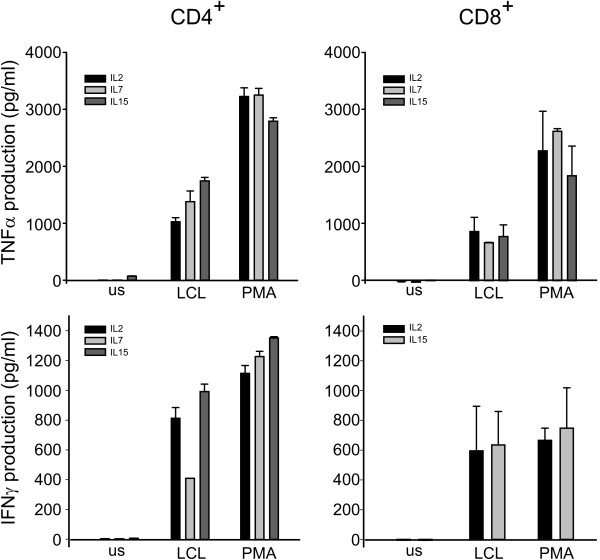
**Th1 cytokine production**. Figure shows TNFα and IFNγ production by CD4+  (left panel) and CD8+ (right panel) T cells in response to stimulation  with autologous LCL or PMA-ionomycin, or unstimulated (us), as assessed by ELISA test. Figure shows mean ± SD of 3 replicate cultures from 2 donors.

Next, we investigated the production of cytokines by cultures in response to different stimuli, such as autologous LCL and PMA-ionomycin, to verify whether the conditions tested have an impact on cytokine production. In particular, we studied the production of Th1 cytokines, namely IFNγ and TNFα, which play an important role in anti-tumor immunity [18,19]. We found that IL-2, IL-7, and IL-15 CD8^+ ^T cell cultures produced comparable amounts of IFNγ and TNFα in response to both stimuli (Figure 3). Moreover, while IL-2, IL-7, and IL-15 CD4^+ ^T cells did not display relevant differences in the amount of TNFα secreted, IL-2 and IL-15 CD4^+ ^T cells produced a higher amount of IFNγ in response to LCL stimulation in comparison to IL-7 cultures, but comparable levels in response to PMA-ionomycin (Figure 3). Cytokine production by IL-21 T cells could not be assessed due to the low number of lymphocytes obtained in cultures.

### Analysis of *in vitro *functional activity

**Figure 4:**
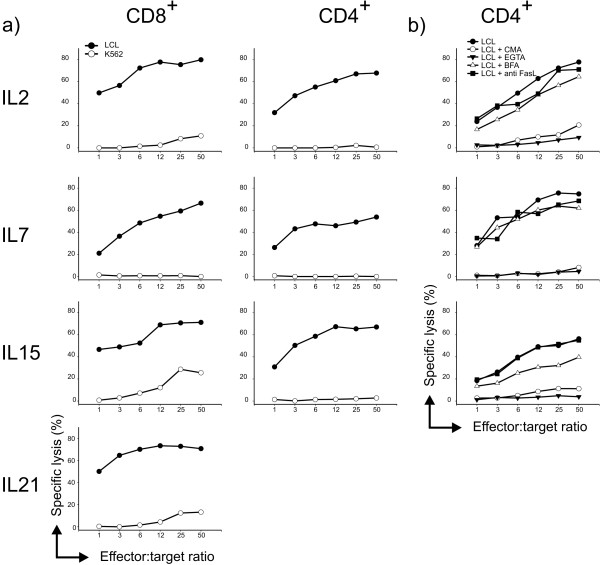
**Lytic activity of EBV-specific CD8^+ ^and CD4^+ ^T cells**. A) Cytotoxic activity was tested by standard 4 h ^51^Cr-release assay in the presence of "cold" K562 at a 5:1 ratio of "cold": "hot" target. B) Lytic mechanisms involved in cytotoxicity. CD4^+ ^T cell line cytotoxicity was evaluated in the presence of CMA and EGTA that block perforin-based pathway, and BFA and anti-FasL mAb that interfere with Fas/FasL-based pathway. Figure shows mean values from 3 independent experiments carried out for each donor cell line.

*In vitro *functional activity was assessed both in short-term and long-term assays. Standard cytotoxicity tests were performed with T cell lines at 21 days of culture. At this time point (third restimulation, see Figure 1), we could test all the cell lines obtained but IL-21 CD4^+ ^T cells. Although NK cell presence was negligible (< 1%), nevertheless all tests were carried out in the presence or absence of an excess of "cold" K562 to eliminate any possible influence of NK-like activity. As shown in Figure 4a, the addition of different cytokines did not modify the lytic activity of either CD8^+ ^or CD4^+ ^T cells. Notably, in contrast with recently published data [7], IL-21-cultured CD8^+ ^T cells showed a strong lytic activity similar to that of cognate IL-2 cultures. To assess the mechanisms involved in lytic activity we focused on CD4^+ ^T cells, as no clear preferential use of granule exocytosis or apoptosis induction is described for this subset. By using compounds that selectively inhibit perforin-based or Fas/FasL-based pathway, we found that all CD4^+ ^T cells obtained, irrespectively of culture conditions, killed their targets through the cytotoxic granule content release (Figure 4b). These findings are in line with our previous observations [13] and the vast majority of data related to EBV-specific cultures [20]. Once again, cytokines used in cultures did not modify functional activity.

**Figure 5:**
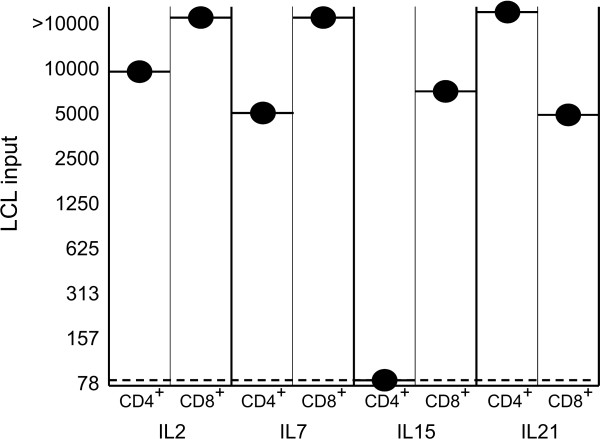
**Inhibition of LCL outgrowth by EBV-specific CTL cultured with different cytokines**. Results are expressed as the minimum LCL number required for successful outgrowth at day 28 of culture (black circles). These values are compared with the corresponding results for outgrowth of LCL seeded without effector T cells (dotted line). Figure shows mean values from 3 independent experiments performed for each donor cell line.

Although commonly used to evaluate functionality of effector T cells, the cytotoxic activity does not always correlate with *in vivo *efficacy, as recently demonstrated not only in mouse models [4] but also in clinical trials [21]. After adoptive transfer, a clue characteristic is the capacity of effector cells to perform sequential killings before exhaustion. As this issue can not be adequately addressed in a short-term test, we performed outgrowth assays that evaluate the ability of a fixed input of T cells to inhibit long-term growth of different numbers of target cells, without the addition of cytokines. This experimental design closely resembles *in vivo *adoptive transfer protocols, which are based on a single infusion of effector T cells without exogenous cytokine supply [13,22]. In both cases, T cells do not likely survive longer than a few days, when they can display their killing potential. Thus, the extent of target elimination could be predictive of the outcome: even few surviving tumor cells can ultimately lead to a successful microculture outgrowth or to the death of the engrafted animals. Due to the low number of cells required (as few as 0.32 × 10^6 ^cells for each test), in this case we could test every cell line obtained. In line with our previous results (data not shown), IL-2-cultured CD8^+ ^T cells disclosed a superior ability to inhibit long-term growth of target cells in comparison to their CD4^+ ^T cell counterpart; a similar trend was observed for CD8^+ ^T lymphocytes cultured in IL-7 or IL-15. Instead, the reverse was true for CD8^+ ^T cells supplied with IL-21. Finally, striking was the finding that IL-15 CD4^+ ^T cells, despite a vigorous *in vitro *cytotoxic activity in short-term assay, did not exert any inhibitory potential (Figure 5).

## Discussion

Recent advances in immunotherapeutic approaches have highlighted the importance of infusing antigen-specific T cells that have ideally a poorly differentiated phenotype and are characterized by a strong proliferative potential upon *in vivo *transfer. These conditions have been partially met by acting on recipient patients with lymphodepleting strategies or by proposing the shortening of T cell *in vitro *expansion protocols with the use of "less differentiating" cytokines. With regard to this latter issue, we exploited a protocol successfully used in immunotherapeutic approaches for EBV-related malignancies to compare the impact of different γ-chain cytokines on phenotype and functionality of cultured T cells, as suggested by recent studies [4,7,8]. We analyzed purified CD8+ and CD4+ T cells to avoid potential  influence of one population on the other one; indeed, despite a trend  toward a "natural" expansion of CD8+T cells, the percentage of CD4+T cells in cultures turns out to be quite various among different  donors and different preparations from the same donor. Our choice furthermore took into account the increasing attention paid on the CD4^+^T cells as actual effector cells in immunotherapeutic approaches [23,24].

Intriguingly, the results presented herein are profoundly different from those of recently published studies. Previous reports, in fact, mainly rely on murine T cells derived from mice expressing transgenic TCR specific for the antigen of interest. All T cells have therefore the desired specificity and hence they only need to be activated *in vitro*, bypassing a potentially long selection phase. Conversely, this phase was absolutely required by our protocol, and covered the first 14 days of culture. Moreover, our protocol envisages the addition of cytokine only after this phase. During this gap, EBV-specific T cells that are present in PBMC of seropositive donors respond to the viral antigens presented by LCL, very likely producing IL-2 that in turn can influence the culture. In this regard, IL-21 has been reported to be capable of reverting the IL-2-induced differentiation [7], but no information is available for IL-7 and IL-15. In addition, it must be noted that *in vitro *expansion selectively involved EBV-specific precursors belonging to the memory compartment and therefore the obtainment of less differentiated cells is expected to be difficult. The long and likely confounding selection phase could be bypassed by performing faster (e.g., overnight) peptide mix stimulation followed by immunomagnetic isolation of cytokine-producing T cells, as recently proposed [25], or by introducing the wanted antigen specificity through CAR- or transgenic TCR-coding vector transduction [26,27]. In these cases, the alternatively chosen cytokines could be added in a less preconditioned milieu, thus driving a less pronounced differentiation of responding T lymphocytes, or, in the case of CAR or TCR transfer, of the whole population of transduced peripheral T cells.

Overall, although the use of γ-chain cytokines other than IL-2 did not produce any substantial *in vitro *improvement, a realistic and clear-cut description of the activity of a determined T cell population should be derived by *in vivo *studies. In this regard, however, we could not produce definitive results since we only had the possibility to test those cultures that reach a sufficient number for infusion. Moreover, the PTLD-SCID mouse model suffers from different intrinsic biases that might have frustrated the purpose of our study. In fact, we have evidence that human T cells survive no longer than 24 hr after *in vivo *transfer [13], even when this follows irradiation or cyclophosphamide treatment of recipient mice. Moreover, this poor survival was verified not only for EBV-specific T cells, but also for less differentiated, CAR-transduced antigen-specific T cells (data not shown). In addition, due to the intrinsic differences between mouse and human adhesion molecules and receptors, it is hard to evaluate the lymph node homing and recirculation capacity that have a fundamental role in the more physiological model described by Gattinoni *et al. *[4], which envisages the transfer of mouse T cells into a syngeneic murine microenvironment. In such experimental context, moreover, the concomitant vaccination strategies make the lymph node homing properties even more relevant, as they dramatically contribute to the improvement of the final outcome [4]. Thus, it is left to be verified in a human context the impact of different lymphoid homing marker expression on the outcome of adoptive transfer strategies.

## Conclusions

As a whole, our results indicate the need to design *ad hoc *protocols to appreciate the impact of γ-chain cytokines other than IL-2 on the functionality of CTL for adoptive cell therapy.

## Competing interests

The authors declare that they have no competing interests.

## Authors' contributions

AM analyzed and interpreted data and wrote the manuscript. RT performed flow cytometry analysis and wrote the manuscript. CT carried out experimental work. PZ and RD critically revised the manuscript. AR conceived the study, and participated in its design and coordination. All authors read and approved the final manuscript.

## References

[B1] Peggs KS Adoptive T cell immunotherapy for cytomegalovirus Expert Opin Biol Ther 2009 9 725 736 10.1517/14712590902967588 19456207

[B2] Rosenberg SA Restifo NP Yang JC Morgan RA Dudley ME Adoptive cell transfer: a clinical path to effective cancer immunotherapy Nat Rev Cancer 2008 8 299 308 10.1038/nrc2355 18354418 PMC2553205

[B3] Gattinoni L Powell DJ Jr Rosenberg SA Restifo NP Adoptive immunotherapy for cancer: building on success Nat Rev Immunol 2006 6 383 393 10.1038/nri1842 16622476 PMC1473162

[B4] Gattinoni L Klebanoff CA Palmer DC Wrzesinski C Kerstann K Yu Z Finkelstein SE Theoret MR Rosenberg SA Restifo NP Acquisition of full effector function in vitro paradoxically impairs the in vivo antitumor efficacy of adoptively transferred CD8+ T cells J Clin Invest 2005 115 1616 1626 10.1172/JCI24480 15931392 PMC1137001

[B5] Dudley ME Yang JC Sherry R Hughes MS Royal R Kammula U Robbins PF Huang J Citrin DE Leitman SF Wunderlich J Restifo NP Thomasian A Downey SG Smith FO Klapper J Morton K Laurencot C White DE Rosenberg SA Adoptive cell therapy for patients with metastatic melanoma: evaluation of intensive myeloablative chemoradiation preparative regimens J Clin Oncol 2008 26 5233 5239 10.1200/JCO.2008.16.5449 18809613 PMC2652090

[B6] Louis CU Straathof K Bollard CM Gerken C Huls MH Gresik MV Wu MF Weiss HL Gee AP Brenner MK Rooney CM Heslop HE Gottschalk S Enhancing the in vivo expansion of adoptively transferred EBV-specific CTL with lymphodepleting CD45 monoclonal antibodies in NPC patients Blood 2009 113 2442 2450 10.1182/blood-2008-05-157222 18971421 PMC2656271

[B7] Hinrichs CS Spolski R Paulos CM Gattinoni L Kerstann KW Palmer DC Klebanoff CA Rosenberg SA Leonard WJ Restifo NP IL-2 and IL-21 confer opposing differentiation programs to CD8+ T cells for adoptive immunotherapy Blood 2008 111 5326 5333 10.1182/blood-2007-09-113050 18276844 PMC2396726

[B8] Hinrichs CS Borman ZA Cassard L Gattinoni L Spolski R Yu Z Sanchez-Perez L Muranski P Kern SJ Logun C Palmer DC Ji Y Reger RN Leonard WJ Danner RL Rosenberg SA Restifo NP Adoptively transferred effector cells derived from naive rather than central memory CD8+ T cells mediate superior antitumor immunity Proc Natl Acad Sci USA 2009 106 17469 17474 10.1073/pnas.0907448106 19805141 PMC2762661

[B9] Kaneko S Mastaglio S Bondanza A Ponzoni M Sanvito F Aldrighetti L Radrizzani M La Seta-Catamancio S Provasi E Mondino A Nagasawa T Fleischhauer K Russo V Traversari C Ciceri F Bordignon C Bonini C IL-7 and IL-15 allow the generation of suicide gene-modified alloreactive self-renewing central memory human T lymphocytes Blood 2009 113 1006 1015 10.1182/blood-2008-05-156059 18978209

[B10] Rooney CM Smith CA Ng CY Loftin S Li C Krance RA Brenner MK Heslop HE Use of gene-modified virus-specific T lymphocytes to control Epstein-Barr-virus-related lymphoproliferation Lancet 1995 345 9 13 10.1016/S0140-6736(95)91150-2 7799740

[B11] Rosato A Milan G Collavo D Zanovello P DNA-based vaccination against tumors expressing the P1A antigen Methods 1999 19 187 190 10.1006/meth.1999.0844 10525455

[B12] Long HM Haigh TA Gudgeon NH Leen AM Tsang CW Brooks J Landais E Houssaint E Lee SP Rickinson AB Taylor GS CD4+ T-cell responses to Epstein-Barr virus (EBV) latent-cycle antigens and the recognition of EBV-transformed lymphoblastoid cell lines J Virol 2005 79 4896 4907 10.1128/JVI.79.8.4896-4907.2005 15795275 PMC1069546

[B13] Merlo A Turrini R Bobisse S Zamarchi R Alaggio R Dolcetti R Mautner J Zanovello P Amadori A Rosato A Virus-Specific Cytotoxic CD4+ T Cells for the Treatment of EBV-Related Tumors J Immunol 2010 184 5895 902 10.4049/jimmunol.0902850 20385879

[B14] Zeng R Spolski R Casas E Zhu W Levy DE Leonard WJ The molecular basis of IL-21-mediated proliferation Blood 2007 109 4135 4142 10.1182/blood-2006-10-054973 17234735 PMC1885510

[B15] Kaka AS Shaffer DR Hartmaier R Leen AM Lu A Bear A Rooney CM Foster AE Genetic modification of T cells with IL-21 enhances antigen presentation and generation of central memory tumor-specific cytotoxic T-lymphocytes J Immunother 2009 32 726 736 10.1097/CJI.0b013e3181ad4071 19561536 PMC2790367

[B16] Kinter AL Godbout EJ McNally JP Sereti I Roby GA O'Shea MA Fauci AS The common gamma-chain cytokines IL-2, IL-7, IL-15, and IL-21 induce the expression of programmed death-1 and its ligands J Immunol 2008 181 6738 6746 18981091 10.4049/jimmunol.181.10.6738

[B17] Vanhoutte VJ McAulay KA McCarrell E Turner M Crawford DH Haque T Cytolytic mechanisms and T-cell receptor Vbeta usage by ex vivo generated Epstein-Barr virus-specific cytotoxic T lymphocytes Immunology 2009 127 577 586 10.1111/j.1365-2567.2008.03035.x 19604308 PMC2729535

[B18] Tannenbaum CS Hamilton TA Immune-inflammatory mechanisms in IFNgamma-mediated anti-tumor activity Semin Cancer Biol 2000 10 113 123 10.1006/scbi.2000.0314 10936062

[B19] Knutson KL Disis ML Tumor antigen-specific T helper cells in cancer immunity and immunotherapy Cancer Immunol Immunother 2005 54 721 728 10.1007/s00262-004-0653-2 16010587 PMC11032889

[B20] Sun Q Burton RL Lucas KG Cytokine production and cytolytic mechanism of CD4(+) cytotoxic T lymphocytes in ex vivo expanded therapeutic Epstein-Barr virus-specific T-cell cultures Blood 2002 99 3302 3309 10.1182/blood.V99.9.3302 11964297

[B21] Haque T Wilkie GM Jones MM Higgins CD Urquhart G Wingate P Burns D McAulay K Turner M Bellamy C Amlot PL Kelly D MacGilchrist A Gandhi MK Swerdlow AJ Crawford DH Allogeneic cytotoxic T-cell therapy for EBV-positive posttransplantation lymphoproliferative disease: results of a phase 2 multicenter clinical trial Blood 2007 110 1123 1131 10.1182/blood-2006-12-063008 17468341

[B22] Lacerda JF Ladanyi M Louie DC Fernandez JM Papadopoulos EB O'Reilly RJ Human Epstein-Barr virus (EBV)-specific cytotoxic T lymphocytes home preferentially to and induce selective regressions of autologous EBV-induced B cell lymphoproliferations in xenografted C.B-17 scid/scid mice J Exp Med 1996 183 1215 1228 10.1084/jem.183.3.1215 8642263 PMC2192329

[B23] Quezada SA Simpson TR Peggs KS Merghoub T Vider J Fan X Blasberg R Yagita H Muranski P Antony PA Restifo NP Allison JP Tumor-reactive CD4+ T cells develop cytotoxic activity and eradicate large established melanoma after transfer into lymphopenic hosts J Exp Med 2010 207 637 650 10.1084/jem.20091918 20156971 PMC2839156

[B24] Xie Y Akpinarli A Maris C Hipkiss EL Lane M Kwon EK Muranski P Restifo NP Antony PA Naive tumor-specific CD4+ T cells differentiated in vivo eradicate established melanoma J Exp Med 2010 207 651 667 10.1084/jem.20091921 20156973 PMC2839147

[B25] Moosmann A Bigalke I Tischer J Schirrmann L Kasten J Tippmer S Leeping M Prevalsek D Jaeger G Ledderose G Mautner J Hammerschmidt W Schendel DJ Kolb HJ Effective and long-term control of EBV PTLD after transfer of peptide-selected T cells Blood 2010 115 2960 70 10.1182/blood-2009-08-236356 20103780

[B26] Vera J Savoldo B Vigouroux S Biagi E Pule M Rossig C Wu J Heslop HE Rooney CM Brenner MK Dotti G T lymphocytes redirected against the kappa light chain of human immunoglobulin efficiently kill mature B lymphocyte-derived malignant cells Blood 2006 108 3890 3897 10.1182/blood-2006-04-017061 16926291 PMC1895462

[B27] Bobisse S Rondina M Merlo A Tisato V Mandruzzato S Amendola M Naldini L Willemsen RA Debets R Zanovello P Rosato A Reprogramming T lymphocytes for melanoma adoptive immunotherapy by T-cell receptor gene transfer with lentiviral vectors Cancer Res 2009 69 9385 9394 10.1158/0008-5472.CAN-09-0494 19996290

